# SapTrap vectors for introducing point mutations with *unc-119+* selection

**DOI:** 10.17912/DDVH-BG64

**Published:** 2018-07-29

**Authors:** Matthew Schwartz, Erik Jorgensen

**Affiliations:** 1 Department of Biology and Howard Hughes Medical Institute, University of Utah, Salt Lake City, Utah 84112-0840

**Figure 1:**
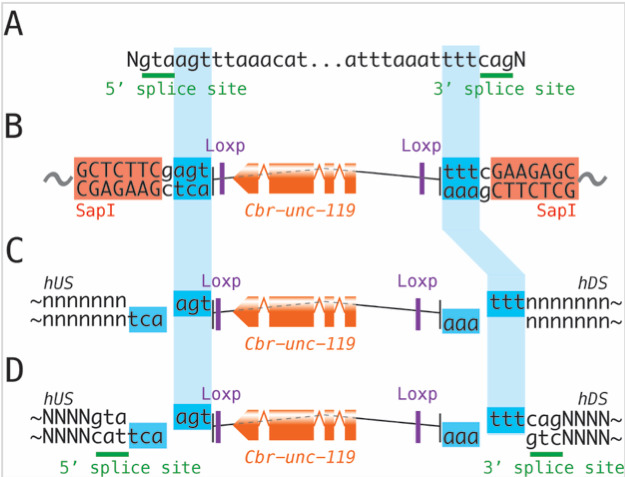
(A) The original syntron sequence used for the *unc-119+* syntron. (B) SapTrap vector (pMLS253) containing the *unc-119+* selectable marker embedded in intron sequences. The *unc-119+* gene is from *C. briggsae*. SapI is a type II restriction enzyme and cuts any DNA sequence adjacent to the binding site (orange), and thereby can generate a variety of 3bp cohesive ends (blue). The cohesive ends correspond to the sequences immediately adjacent to the 5’ and 3’ splice-sites in the parent syntron. pMLS415 is identical except it contains Lox2272 sites instead of LoxP sites. (C) To embed the *Cbr-unc-119+* marker in a native intron, simply add compatible cohesive ends to the homology regions (hUS and hDS) of the repair template. (D) To create a new syntron within a coding region, additionally add 5’ and 3’ slice sites adjacent to the cohesive ends. ‘N’s represent coding bases, ‘n’s represent intronic bases, ‘…’ represents bases not shown.

## Description

SapTrap assembly generates CRISPR targeting vectors using a Golden Gate cloning strategy (Schwartz and Jorgensen, 2016). The original toolkit was designed for inserting tags, such as fluorescent proteins or affinity tags, into protein coding genes in *C. elegans*. In the original design, the floxed *unc-119+* selectable marker was incorporated into the tag. For site-directed mutagenesis, the selectable marker is useful but a tag is unnecessary. We have developed a new set of vectors dedicated for introducing point mutations using *unc-119+* selection. These new vectors contain only a floxed *unc-119+* selectable marker in the reverse orientation of a synthetic intron (‘syntron’) that lacks both 5’ and 3’ splice sites. This *unc-119+* module can be inserted directly into a native intron. Alternatively, splice site sequences can be appended for insertion into a coding sequence to generate a syntron in the coding sequence. The desired point mutations are designed into the homology arms of the selection construct. SapTrap is used to assemble a plasmid encoding the guide RNA and the template. After injection and selection of *unc-119*+ animals, the selectable marker can be removed by expression of Cre recombinase to generate an animal with only the desired point mutation and a single Lox site in the gene of interest.

Reagents for point mutation vectors differ from reagents for tagging vectors in three ways:

(1) Different cohesive-end sequences are used to join the homology arms to the marker module.

(2) Splice-site sequences must be added to the homology arms if inserting into a coding region.

(3) The point mutation, the guide RNA cut site, and selectable marker must be carefully arranged. Specifically, point mutations must be positioned between the cut site and the selectable marker. This arrangement ensures that all Unc-119+ animals have incorporated the desired point mutations. Homology-directed repair fixes double stranded breaks by copying the repair template DNA across the break. The extent of copying beyond the immediate vicinity of the break is stochastic. By selecting for Unc-119+ incorporation, we select for repair events that copy the repair template between the break and the *unc-119+* cassette. If the point mutation were placed beyond the *unc-119+* cassette, then repair could drop off of from the repair template before encountering the mutation, thereby failing to fix the mutation in the genome. Then some Unc-119+ animals would lack the mutation, resulting in significant numbers of false-positive strains.

We recently released the SapTrap Builder software, a desktop utility for designing reagents for SapTrap assemblies (Schwartz and Jorgensen, 2018). To generate reagents for introducing point mutations using the new selectable marker-only vectors described here, simply select ‘Native Intron’ or ‘Syntron’ in the ‘Tag Type’ field during design. SapTrap Builder will add the correct cohesive ends to the homology arms, add splice-site sequences when needed, and monitor for the arrangement of the cut site, marker insertion site and mutations, alerting the user to unfavorable arrangements.

## Reagents

Plasmids are available through Addgene.

**Table:** 

**Plasmid**	**Insert**	**Description**	**Addgene Number**
**pMLS253**	syntron:LoxP-*Cbr-unc-119*-LoxP	LoxP point mutation marker donor	114271
**pMLS415**	syntron:Lox2272-*Cbr-unc-119*-Lox2272	Lox2272 point mutation marker donor	114272
